# Exploring Trehalose on the Release of Levonorgestrel from Implantable PLGA Microneedles

**DOI:** 10.3390/polym12010059

**Published:** 2020-01-01

**Authors:** Xiaoyu Zhao, Suohui Zhang, Guozhong Yang, Zequan Zhou, Yunhua Gao

**Affiliations:** 1 Key Laboratory of Photochemical Conversion and Optoelectronic Materials, Technical Institute of Physics and Chemistry, Chinese Academy of Sciences, Beijing 100190, China; xyzhao@mail.ipc.ac.cn (X.Z.); suohuizhang@mail.ipc.ac.cn (S.Z.); yangguozhong@mail.ipc.ac.cn (G.Y.); zhouzequan16@mails.ucas.edu.cn (Z.Z.); 2 University of Chinese Academy of Sciences, Beijing 100049, China

**Keywords:** poly (lactic-co-glycolic acid), microneedles, drug delivery system, trehalose

## Abstract

Hydrophobic drugs wrapped in poly (lactic-co-glycolic acid) (PLGA)-based microneedles (MNs) require a long time to release completely. To obtain the desired duration, it is still necessary to modulate the release of hydrophobic drugs from MNs, while the PLGA composition is unchangeable. In this work, implantable PLGA microneedles (IPMNs) composed of PLGA arrowheads encapsulating levonorgestrel (LNG) and a water-soluble supporting array were designed. We explored trehalose used as a porogen on the release of hydrophobic LNG from PLGA-based MNs. Varying the trehalose content in PLGA arrowheads could induce different rates of drug release. The highest cumulative release of LNG was 76.2 ± 3.9% for IPMNs with 33.3% trehalose during 21 days in vitro, while the cumulative release of LNG was 60.4 ± 3.5% for IPMNs without trehalose. Pharmacokinetic results in rats showed that plasma levels of LNG were sustained for 13 days for IPMNs with 33.3% trehalose and 16 days for IPMNs without trehalose. Furthermore, the PLGA arrowheads with trehalose degraded more rapidly than those without trehalose over 21 days in rats. Consequently, using trehalose as a porogen was a feasible approach to modulate the release of a hydrophobic drug from PLGA-based MNs.

## 1. Introduction

Polymeric microneedles (MNs) are extensively used in controlled-release drug delivery through a minimally-invasive strategy [1,2,3]. Since receiving approval by the Food and Drug Administration, poly (lactic-co-glycolic acid) (PLGA) has attracted substantial attention as a drug delivery carrier for polymeric MNs due to its biocompatibility, biodegradability, and favorable mechanical performance [4,5,6]. The melting and solvent casting methods are usually used to fabricate PLGA-based MNs. However, a high temperature (often greater than 140 °C) is required in the melting method, which may decrease the activity of thermosensitive drugs [7,8,9]. As an alternative strategy, the solvent casting method has been intensively investigated. Organic solvents (e.g., dichloromethane, dioxane, tetrahydrofuran, dimethylformamide, diglyme, etc.) are generally employed as vehicles to dissolve PLGA and drugs at room temperature [10,11,12,13]. The fabrication of bubble-microneedle patches encapsulating levonorgestrel (LNG) using dioxane and tetrahydrofuran to dissolve PLGA and polylactic acid has been reported. These MNs can rapidly implant into the skin within 5 s and slowly release the LNG for contraception [12].

The drug release rate of PLGA-based systems is influenced by many factors, such as PLGA composition, the size and shape of devices, the drug type, and drug release medium [14]. Among these factors, the hydrophilicity/hydrophobicity of the drug plays a key role in the rate of drug delivery [15]. Liu et al. [16] reported the different release behaviors of hydrophilic and hydrophobic drugs from PLGA 50/50 films. When the drug was released completely, the release period of hydrophobic sirolimus was almost 40 days. This cycle was approximately twice as long as the period observed from hydrophilic ciprofloxacin hydrochloride. Hydrophobic drugs often display a slower release than hydrophilic drugs in PLGA matrices [17,18]. This same issue also exists in PLGA-based MNs [12]. Therefore, it is necessary to accelerate the release of hydrophobic drugs from PLGA-based MNs to obtain the expected release duration. It will be helpful to broaden the application of PLGA-based MNs encapsulating hydrophobic drugs in vivo. Many methods have been reported to expedite the release rate of hydrophobic drugs, such as the use of porous matrices, the potential role of excipients, and enhanced drug loading [19]. Among them, increasing the porous structure of polymer devices is a simple path [15]. It has been reported that lidocaine (which is poorly soluble in water) possesses a faster release profile from porous PLGA microparticles than from nonporous PLGA microparticles [20]. Porogens have been widely used to create porosity in PLGA matrices [21,22,23,24,25]. Therefore, it is of great significance to explore the role of porogens in the release of hydrophobic drugs from PLGA-based MNs.

**Figure 1:**
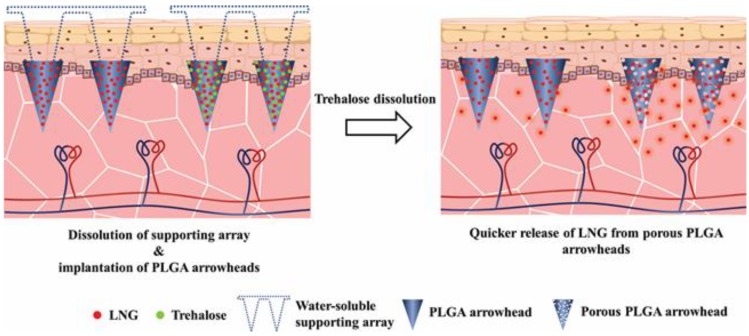
Schematic illustration of the release of LNG from implantable PLGA microneedles with or without trehalose.

In our study, implantable PLGA microneedles (IPMNs) were fabricated by integrating the drug-loaded PLGA arrowheads and a water-soluble supporting array. LNG, a potent progestogen with high hydrophobicity (Log P = 3.88), was selected as the model drug [26,27]. *N*-methyl-2-pyrrolidone (NMP), a biocompatible solvent with strong solvating ability [28,29,30], was chosen for the PLGA arrowhead formulation. Trehalose can fully dissolve in NMP as well as water, which allows it to be well dispersed in the PLGA matrix and then leave holes in PLGA arrowheads after exposure to water. Therefore, trehalose was selected as a porogen; a schematic illustration is shown in Figure 1. Once inserted, LNG-loaded PLGA arrowheads were implanted into the skin after the water-soluble supporting array dissolved. The porous PLGA arrowheads were created by the dissolution of trehalose in the interstitial fluid in the skin, resulting in a quicker release of hydrophobic LNG. In this paper, the skin penetration capability, insertion depth, and dissolution performance of IPMNs were studied. Moreover, effects of trehalose on both the LNG release from IPMNs and the degradability of PLGA arrowheads were investigated.

## 2. Materials and Methods

### 2.1. Materials and Subjects

PLGA (RESOMER^®^ RG 502, inherent viscosity 0.16–0.24 dL/g) was purchased from Evonik Industries AG (Darmstadt, Germany). Polyvinyl alcohol (PVA, MW approximately 30,000) was purchased from Jiangxi Alpha Hi-Tech Pharmaceutical Co., Ltd. (Pingxiang, China). Polyvinyl pyrrolidone (PVP)-k120 was acquired from Shanghai Gobekie New Material Technology Co., Ltd. (Shanghai, China). LNG was purchased from Qinhuangdao ZiZhu Pharmaceutical Co., Ltd. (Qinhuangdao, China). Standard substances of LNG and canrenone were procured from the National Institutes for Food and Drug Control (Beijing, China). Trehalose (dehydrate) was purchased from Hayashibara (Okayama, Japan). Polydimethylsiloxane (PDMS, Sylgard 184) was obtained from Dow Corning (Midland, MI, USA). Chemically pure grade polyethylene glycol 400 was obtained from Sinopharm Chemical Reagent Co., Ltd. (Shanghai, China). Acetonitrile and methanol, both of high performance liquid chromatography (HPLC) grade, purchased from Sigma (St. Louis, MO, USA). High performance liquid chromatography–tandem mass (LC/MS) grade formic acid was obtained from Fisher Scientific (Fair Lawn, NJ, USA). Analytical grade NMP and HPLC grade hexamethylene were both purchased from Tianjin Heowns Biochemical Technology Co., Ltd. (Tianjin, China).

Female Sprague Dawley rats (7 weeks old, 200 ± 15 g) were purchased from Beijing Jinmuyang Experimental Animals Co., Ltd (Beijing, China). All rats were housed under specific pathogen-free conditions. All animal experiments were conducted following the Guide for the Care and Use of Laboratory Animals (Eighth edition, 2011).

### 2.2. Fabrication of IPMNs

The IPMNs consisted of biodegradable PLGA arrowheads and water-soluble MNs. PLGA arrowheads encapsulating drugs acted as drug depots, and water-soluble MNs served as a supporting array. 

**Figure 2:**
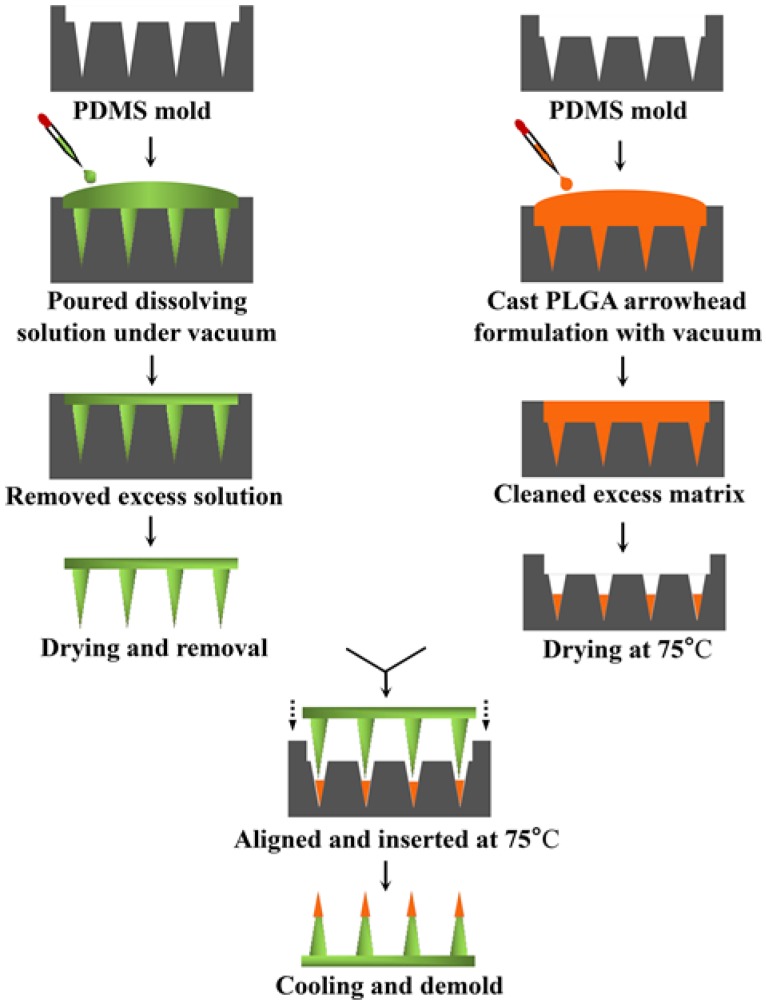
Schematic of IPMNs fabrication process.

The IPMN fabrication process is summarized in Figure 2. A dissolvable solution consisting of 10% (w/w) PVA and 20% (w/w) PVP-k120 was cast in a PDMS mold. MN cavities were filled with a dissolvable matrix under vacuum for 5 min. Then, the excess matrix on the outside of the mold was cleared away using a plastic plate. Next, the mold was placed under 30% relative humidity at room temperature (RT) for 4 h. Then, water-soluble MNs were acquired by removing them from the mold.

For the sake of making PLGA arrowheads without trehalose, the PLGA matrix containing the drug was obtained by dispersing 15% (w/w) solids in NMP. The solids were composed of PLGA and LNG with different mass ratios (7.5:1, 5:1, 3:1, and 3:2). Then, keeping the mass ratio of PLGA:LNG at 3:2, 16.6% and 33.3% (w/w based on PLGA amount) trehalose were added to the PLGA matrix to obtain two arrowhead formulations. To observe PLGA arrowheads visually, an oil-soluble fluorescent red (FR) dye was selected to replace LNG. A series of arrowhead formulations without the drug were synthesized according to the parameters given in Table S1. The ingredients (i.e., PLGA, LNG, trehalose, and FR) in the amounts mentioned in this paper all dissolved completely in NMP.

PLGA arrowheads were manufactured by pouring the PLGA arrowhead formulation onto the PDMS mold. The mold cavities were then held under vacuum to remove bubbles. Afterwards, the superfluous matrix was swept off by using a plastic plate. The mold was moved to a 75 °C oven for 3 h to evaporate the solvent and solidify the needles. Then, the mold was transferred to a heating plate at 75 °C, which was hot enough to melt PLGA arrowheads. Next, water-soluble MNs were aligned and inserted into the mold cavities filled with molten PLGA arrowheads. After cooling rapidly, the IPMNs were obtained by separation from the mold. The morphology of the IPMNs was examined using a stereomicroscope (SMP1000, Nikon, Japan), and side views were obtained using an optical microscope (BX51, Olympus, Japan). Despite the high boiling point (202 °C) of NMP, it is sufficient to endow the IPMNs with a skin penetration capability by evaporating NMP at 75 °C for 3 h. It is possible that very little solvent remained in PLGA arrowheads, which does not affect their mechanical performance.

### 2.3. Skin Penetration and Insertion Depth Tests

To evaluate the skin penetration capability and separability of IPMNs in vitro, FR-loaded IPMNs (PLGA arrowhead formulation 1 in Table S1) were inserted into porcine ear skin using a homemade applicator (30 N/cm^2^) for 20 s. The skin after treatment of IPMNs was placed on an agarose hydrogel (3% w/w) to keep it moist for 20 min. Then, the IPMNs were removed. The skin and the residual FR-loaded IPMNs were photographed with a stereomicroscope. To further evaluate the insertion capability and separability of LNG-loaded IPMNs (PLGA and LNG with a mass ratio of 3:2) with 33.3% trehalose, a homologous experiment was carried out. Then, the skin was photographed after insertion with a stereomicroscope.

To evaluate the insertion depth, a confocal laser scanning microscope (CLSM, A1R MP, Nikon, Japan) was used to observe the skin after the FR-loaded IPMNs were inserted. Fluorescence of the FR dye was received at an excitation wavelength of 530 nm. Scanning was conducted once at an interval of 20 μm from the original fluorescence of the skin surface through the z-axis vertical to the xy-plane. To observe PLGA arrowhead implantation in the skin visually, 3D confocal reconstruction images were acquired by the xyz stack.

### 2.4. Determination of Mechanical Performance of IPMNs

A force-displacement test station (1220SB, SA Precision Instruments Corporation, Ltd., Dongguan, China) was used to measure the mechanical performance of LNG-loaded IPMNs. Three types of IPMNs were composed of PLGA and LNG at a mass ratio of 3:2, but with different contents of trehalose (0%, 16.6%, and 33.3%). The experimental procedures and conditions described in [31] were adopted in this test.

### 2.5. Quantification of LNG Loaded into IPMNs

To determine the amount of LNG loaded into IPMNs, one piece of IPMN was dispersed in 3.5 mL of acetonitrile, followed by vortex mixing. Then, 1.5 mL of distilled water was added to the sample, followed by mixing with a vortexer. The supernatant was detected by HPLC after centrifugation for 10 min at 10,000 r/min. The drug loading of IPMNs with different PLGA arrowhead formulations was shown in Table S2.

Using an acetonitrile-water mixture (70:30 v/v) as the mobile phase with a flow rate of 1.0 mL/min, HPLC (LC-20AT, Shimadzu, Tokyo, Japan) was used to analyze LNG. A YMC-Triart C18 (YMC Co., Ltd., Kyoto, Japan) column with a particle size of 5 μm (250 × 4.6 mm i.d.) was used at 30 °C. The injection volume was 20 μL, and LNG was detected at 240 nm. The peak area of LNG was used as a quantification criterion according to the external standard method, and a standard calibration curve was constructed over a linear range of 0.1–20 μg/mL, with a correlation coefficient ≥ 0.9999.

### 2.6. In Vitro Dissolution of IPMNs and Delivery to Skin

LNG-loaded IPMNs without trehalose consisting of PLGA and LNG with a mass ratio of 3:2 were selected as test objects. IPMNs were applied to porcine ear skin with a force of 30 N/cm^2^ for 20 s using a homemade applicator. The skin after treatment was kept on the hydrogel for various times (0, 3, 5, 10, 20, and 30 min); then, the IPMNs were peeled off at each time point. Morphological changes in the IPMNs after attachment for different times were observed using an optical microscope. Simultaneously, the residual amounts of LNG in these IPMNs were detected by HPLC. The process of sample treatment and chromatographic conditions were the same as those described in Section 2.5. The amount of LNG deposited in the skin was determined by subtracting the remaining LNG amount from the initial LNG amount in IPMNs. The percentage of LNG deposited in the skin was obtained by dividing the amount deposited in the skin by the original amount. Finally, the relationship between the percentage of LNG deposited in the skin and application time was evaluated.

### 2.7. Structural Analysis of Compounds in PLGA Arrowheads

To investigate the existence of substances in PLGA arrowheads, a variety of powder samples were characterized by X-ray diffraction (XRD) (D8 focus, Bruker, Karlsruhe, Germany). Then, measurements were recorded from a 2θ angle of 10° to 40°. For each single-component sample (e.g., PLGA, LNG and trehalose), the powder was tested directly without any treatment. For the multicomponent sample, the powder was acquired by heating, to drain the matrix by dissolving PLGA, LNG, and trehalose mixture at a 3:2:1 mass ratio in NMP.

### 2.8. Characterization of Morphology of PLGA Arrowheads

To assess the pore-forming effect of trehalose, two kinds of PLGA arrowhead formulations (formulation 2 and 3 in Table S1) were used to fabricate IPMNs. Two types of IPMNs were dissolved in 3 mL of distilled water, followed by vortex mixing for 10 h at a rate of 500 r/min. The suspension was dropped on a clean silicon wafer and dried at RT. Next, the morphologies of PLGA arrowheads were observed via scanning electron microscopy (SEM) (5 kV, S-4800, Hitachi Ltd., Tokyo, Japan).

### 2.9. In Vitro Release of LNG from IPMNs

The LNG release curves of IPMNs with different PLGA arrowhead formulations (such as different ratios of PLGA to LNG and different contents of trehalose) were accomplished with dialysis bags. IPMNs were placed in a dialysis bag and hung in a conical flask with a dissolving medium (40% polyethylene glycol 400-PBS, pH = 7.4). Then, the conical flask was placed in a 37 °C water bath with magnetic stirring (280 r/min) [31]. Samples were collected from the release medium at specified time intervals and replenished with the same volume of fresh medium. The amount of LNG in each sample was detected by HPLC after centrifuging the sample for 10 min at 10000 r/min. The experimental conditions of HPLC were the same as those described in Section 2.5.

### 2.10. In Vitro Transdermal Delivery of LNG from IPMNs

LNG-loaded IPMNs, composed of PLGA and LNG with a mass ratio of 3:2, were evaluated in this experiment. LNG-loaded IPMNs were prepared with PLGA arrowhead formulations containing 0%, 16.6%, and 33.3% trehalose. In vitro LNG transdermal delivery was processed by modified Franz diffusion cells (912-SCT-S, Logan Instruments Corporation, Somerset, NJ, USA). First, porcine ear skin was soaked in PBS (pH = 7.4) for 1 h [32]. Then, LNG-loaded IPMNs were penetrated into porcine ear skin and were kept on the skin for 20 min. After peeling off the IPMNs, the skin was positioned between the donor and receptor compartments of the Franz cell. The cuticle of the skin faced towards the donor compartment. The receptor compartment contained 2.7 mL of a receiver solution (40% polyethylene glycol 400-PBS, pH = 7.4). Samples were obtained from the receptor compartment and supplied with the same volume of fresh solution once a day until day 21. The concentration of LNG in each sample was measured by HPLC after centrifuging the sample for 10 min at 10,000 r/min. The test conditions for HPLC are described in Section 2.5.

### 2.11. In Vivo Pharmacokinetics

Female Sprague Dawley rats were randomly allocated into three groups with six rats in each: (1) LNG-loaded IPMNs with 33.3% trehalose, (2) LNG-loaded IPMNs without trehalose, (3) subcutaneous injection. For these three groups, 500 μg of LNG was given to each rat. For the IPMNs, the formulations of the PLGA arrowheads were based on the mass ratio of PLGA:LNG at 3:2. Before the experiment, the abdominal hair was removed from the rats. Then, IPMNs were inserted into the abdominal skin with a homemade applicator. After 20 min, the IPMNs were stripped off. The rats in the third group were given a subcutaneous injection of biodegradable LNG matrix using NMP as the medium, comprising 0.75% w/w PLGA, 0.5% w/w LNG and 0.25% w/w trehalose. Blood samples were obtained from the tail veins of the rats at each time point. The plasma was received by centrifugation and then stored at −20 °C before testing.

The LNG levels in rat plasma were determined using ultra performance liquid chromatography–tandem mass spectrometry (UPLC-MS/MS), which included a UHPLC (UltiMate3000, Dionex, Sunnyvale, CA, USA) and an orbitrap mass spectrometer (Q-extractive, Thermo Fisher Scientific, Waltham, MA, USA). Hexamethylene was used as the extracting agent for plasma. After drying the extractant, the residue was redispersed in 100 μL of mobile phase. Chromatographic separation was accomplished on a C18 column (100 mm × 2.1 mm, 3 μm, Hypersil GOLD™, Thermo Fisher Scientific, Waltham, MA, USA) at 30 °C. The mobile phase was composed of methanol and water, with 0.01% formic acid (80:20 v/v). The flow rate was 0.2 mL/min, and the injection volume was 10 μL. Positive ion mode was chosen for electrospray ionization. The MS/MS acquisitions were fulfilled, and target MS^2^ mode was used for quantification. The precursor product ion pairs were m/z 313.2158→109.0650 for LNG and m/z 341.2106→107.0858 for canrenone (internal standard). A standard calibration curve was constructed over the linear range of 0.25–50 ng/mL with a correlation coefficient ≥ 0.9995.

### 2.12. In Vivo Degradation Test of PLGA Arrowheads

To investigate the effect of porogen (trehalose) on the degradation rate of PLGA arrowheads in vivo, two kinds of FR-loaded IPMNs (PLGA arrowhead formulation 4 and 5 in Table S1) were administrated to the rats’ abdominal skin. The experimental method is described in Section 2.11. The rats were sacrificed on day 0 and week1, week 2, and week 3 after administration. Then, the application sites were excised for histological sectioning. The 14 μm-thick frozen skin sections were obtained with a freezing-microtome (NX50, Thermo, Waltham, MA, USA), and the samples were analyzed by a fluorescence microscope (BX51, Olympus, Tokyo, Japan).

### 2.13. Statistical Analysis

Data were described as the mean value ± standard deviation (s.d.). Statistical data were evaluated with ANOVA using IBM SPSS Statistics 21. *p* < 0.05 was considered to be statistically significant.

## 3. Results and Discussion

### 3.1. Design and Fabrication of IPMNs

**Figure 3:**
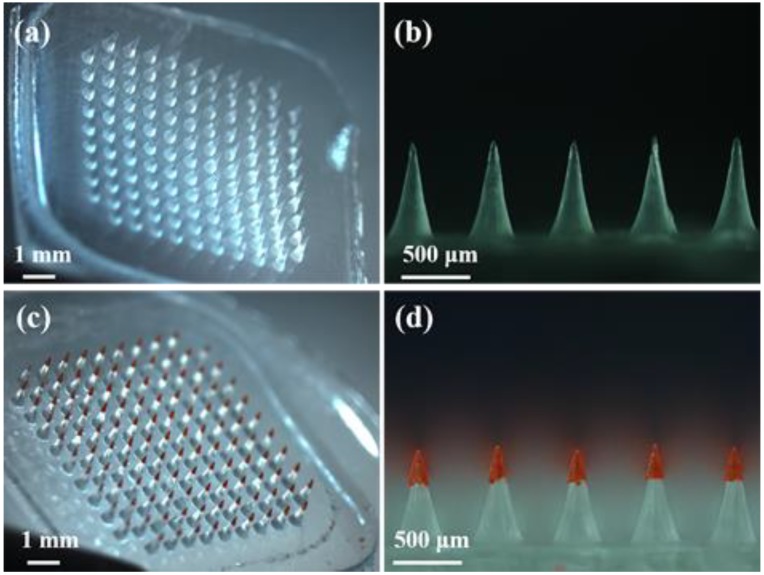
(**a**) Stereomicroscopic image of water-soluble MNs. (**b**) Side view of water-soluble MNs. (**c**) Stereomicroscopic image of FR-loaded IPMNs. (**d**) Side view of FR-loaded IPMNs.

Water-soluble MNs (Figure 3a) were arranged in a 117 (11 × 11 − 4) array in an area of 6 mm × 6 mm. Each MN was conical. To avoid the right angles of the quadrate patches destroying skin, the four MNs in the corners were abandoned. The height and base width of MNs were 700 μm and 320 μm, respectively, with a center-to-center distance of 600 μm (Figure 3b). After integrating the FR-loaded PLGA arrowheads, a stereomicroscopic image of the IPMNs was taken; see Figure 3c. The area and array of IPMNs were the same as those of the water-soluble MNs. Each IPMN, with a height of 750 μm, included a 250 μm PLGA arrowhead with a base width of 140 μm and approximately 200 μm of overlap (Figure 3d).

When IPMNs contacted the tissue fluid in the skin, water-soluble supporting arrays dissolved, and drug-loaded PLGA arrowheads were easily left. PVA and PVP were used in the supporting arrays, which are safe polymer materials with good water solubility and stiff mechanical strength [5,33]. PLGA is usually used as a MN material for sustained release due to its excellent biocompatibility, slow degradability, and remarkable mechanical properties [8,10,34]. PLGA biodegrades into lactic and glycolic acids, and is ultimately cleared as water and carbon dioxide from the body [35]. The effortless separability of PLGA arrowheads, the sustained release of hydrophobic LNG, and no emergence of harmful medical waste can be accomplished by this IPMN system. Considering the hydrophobicity of PLGA [36], the release rate of LNG can be modulated by the addition of water-soluble porogens. Trehalose, a natural disaccharide that includes two glucose molecules, has outstanding water solubility. In addition, it is a safe pharmaceutical excipient [37,38,39]. Because of the good solubility of trehalose in NMP, it could be fully dispersed in the PLGA arrowhead matrix. Moreover, pores in PLGA arrowheads can be easily created, since trehalose dissolves in water. Furthermore, it is easy to cast the PLGA matrix at room temperature because of the high boiling point (202 °C) and less volatile properties of NMP [40,41]. Compared to previous studies on separable MNs [7,42,43], the fabrication of IPMNs in this study is simple, which provides the possibility for mechanized production in the future.

### 3.2. Separability and Insertion Depth

To investigate the penetration capability and separability of IPMNs in vitro, FR-loaded IPMNs were applied to porcine ear skin. After 20 min, IPMNs were peeled off. The whole array of red spots was displayed, demonstrating that IPMNs could successfully be inserted and leave FR-loaded PLGA arrowheads in the skin (Figure S1a). Figure S1b shows that few FR remained in the residual IPMNs, further indicating that PLGA arrowheads were completely embedded into the skin. Therefore, the full separability of PLGA arrowheads from IPMNs was easily achieved in a short time, which avoids wearing for long periods and promotes patient compliance.

The penetration capability and separability of LNG-loaded IPMNs with trehalose were further assessed. As shown in Figure S2, the mechanical strength of LNG-loaded IPMNs presented a declining trend as the trehalose content increased. However, the inset in Figure S2 reveals that LNG-loaded IPMNs with 33.3% trehalose could completely implant PLGA arrowheads into the skin. These results indicate that the amount of trehalose in PLGA arrowheads does not influence the insertion capability and separability of LNG-loaded IPMNs.

**Figure 4:**
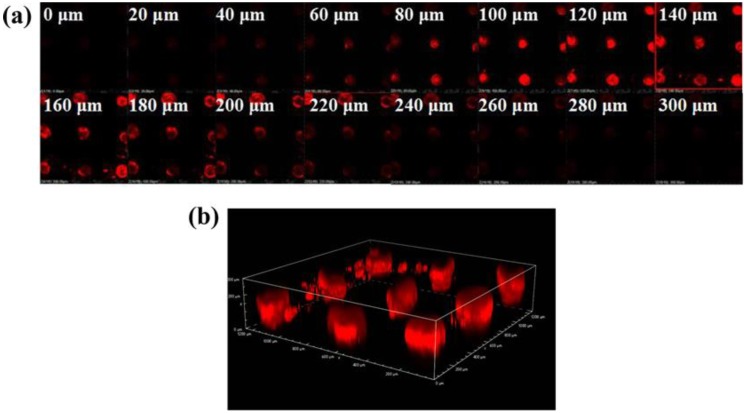
(**a**) Microconduits after the applied FR-loaded IPMNs in the porcine ear skin were scanned through the z-axis visualized by CLSM. (**b**) A 3D image of nine implantation sites created by FR-loaded IPMNs in the porcine ear skin.

To further evaluate the insertion depth of IPMNs, the treated skin was investigated by CLSM analysis. Figure 4a shows that the fluorescence intensity increased initially and gradually lessened after attaining a maximum value at varying depths. The fluorescence intensity disappeared at a depth of 300 μm. Thus, the insertion depth of IPMNs was approximately 300 μm longer than the height of PLGA arrowheads. A 3D image of nine implantation sites created by FR-loaded IPMNs in the porcine ear skin is displayed in Figure 4b, which visually indicates that PLGA arrowheads were completely implanted into the skin as drug depots. These results made it possible to dramatically improve drug utilization [44,45,46].

### 3.3. In Vitro Dissolution Performance of LNG-loaded IPMNs

**Figure 5:**
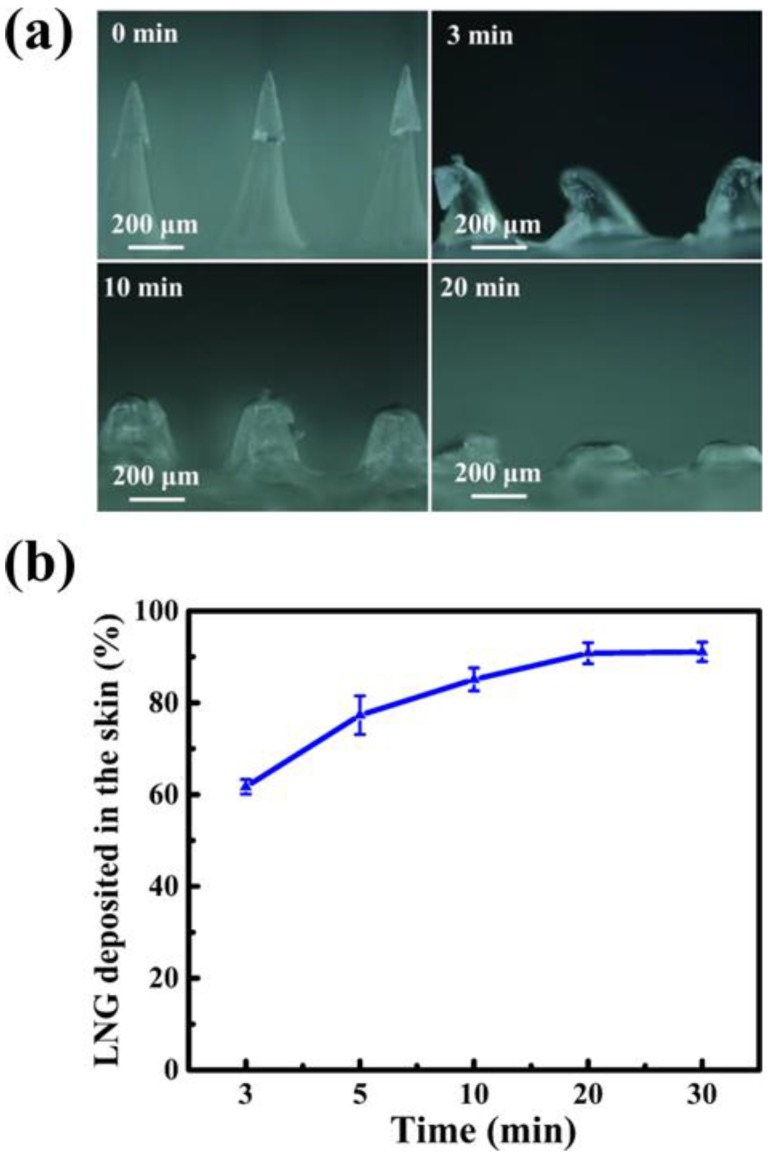
(**a**) Dark field micrographs of LNG-loaded IPMNs after inserted into porcine ear skin in vitro at different application times. (**b**) Percentage of LNG deposited in the porcine ear skin at different application times (mean ± s.d., n = 3). The initial amount of LNG in one piece of IPMNs was 50 μg.

The morphologies of LNG-loaded IPMNs before and after insertion in vitro were investigated through dark field micrographs (Figure 5a). The water-soluble supporting arrays could dissolve in 20 min, leading to the gradual disappearance of water-soluble MNs and the implantation of LNG-loaded PLGA arrowheads into the skin. As shown in Figure 5b, the percentage of LNG deposited in the skin increased with the application time. After attaching for 20 and 30 min, the percentages of LNG deposited in the skin were 90.8 ± 2.3% and 91.1 ± 2.1%, respectively. However, these two groups showed no significant difference (*p* > 0.05). Therefore, 20 min was the optimal application time. This result provided a valuable reference for subsequent in vitro and in vivo experiments.

The dissolution performance of IPMNs is related to the materials of the water-soluble supporting arrays. Different soluble polymers have different dissolution rates [31]. The dissolution rate of IPMNs was constant when water-soluble supporting arrays were identical. Therefore, the dissolution performance of IPMNs was not affected by changes in the PLGA arrowhead formulation.

### 3.4. Structure and Morphology

**Figure 6:**
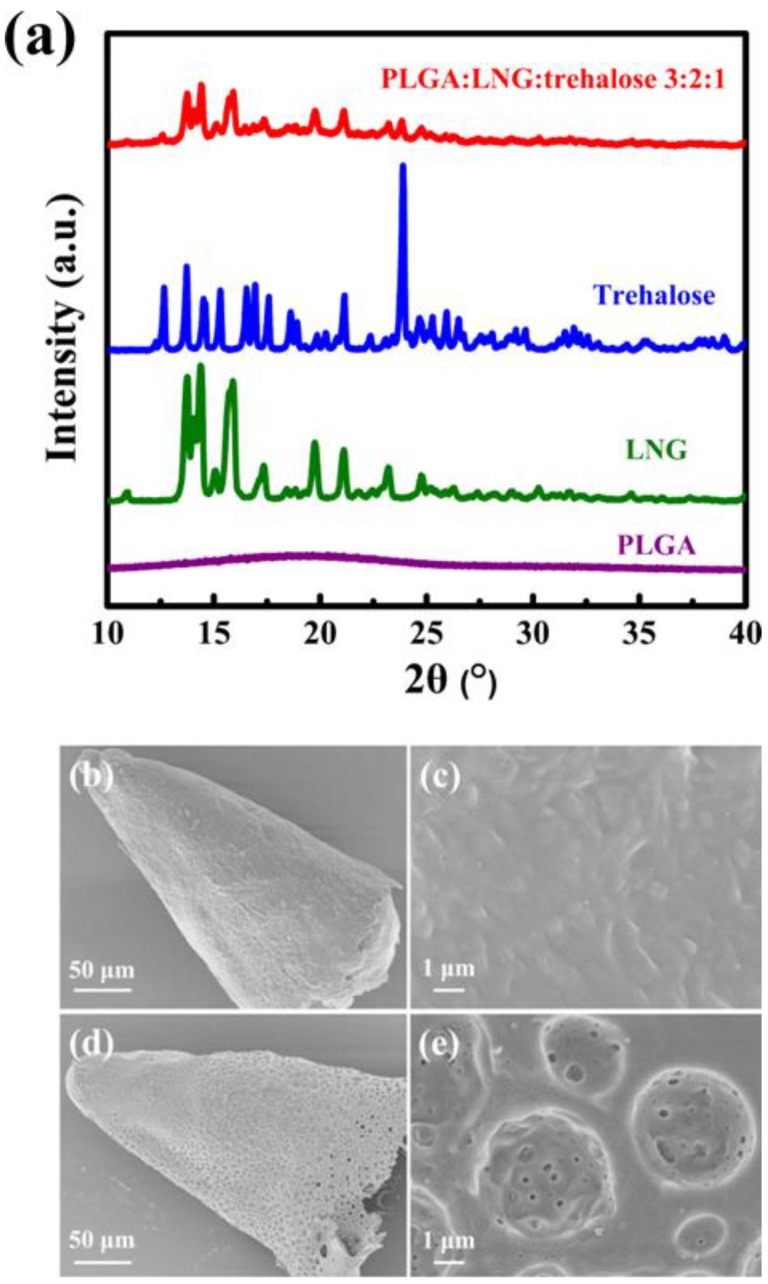
(**a**) XRD patterns of PLGA, LNG, trehalose (dehydrate) and the dried powder of PLGA arrowhead matrix (containing PLGA:LNG:trehalose with a mass ratio of 3:2:1). (**b**–**e**) Morphologies of PLGA arrowheads with or without trehalose after immersion in water: (**b**) PLGA arrowhead without trehalose, (**c**) enlarged picture of (**b**), (**d**) PLGA arrowhead with 33.3% trehalose, (**e**) enlarged image of (**d**).

To investigate the form of all substances present in PLGA arrowheads, XRD was used to analyze the powder samples. XRD patterns of PLGA (RG502), LNG and trehalose (dehydrate) were provided (Figure 6a). The results confirmed that PLGA was amorphous because of the wide peak from 13° to 25°. The XRD patterns of LNG and trehalose (dehydrate) exhibited characteristic diffraction peaks, which were in agreement with the literature [27,47]. The results indicated that LNG and trehalose were respectively present in the crystalline state. Figure 6a also shows that the dried powder of the PLGA arrowhead matrix consisted of amorphous PLGA, crystalline LNG, and trehalose (dehydrate). The results revealed that crystal forms of LNG and trehalose were unchanged during the whole fabrication process, which would not affect the pharmacodynamics of LNG or the properties of trehalose [48,49].

To evaluate the pore-forming ability of trehalose, the morphologies of PLGA arrowheads after immersion in water were observed by SEM (Figure 6b–e). For the PLGA arrowhead without trehalose (Figure 6b), the height was approximately 250 μm, which was in agreement with the side view of PLGA arrowheads from the optical microscopy images (Figure 3d). Moreover, a relatively smooth appearance was observed in the enlarged image (Figure 6c). The height of PLGA arrowhead with 33.3% trehalose was similar to that without trehalose (Figure 6d). However, abundant pores could be observed on the surface of PLGA arrowhead with trehalose. Furthermore, many smaller pores were also clearly observed in each larger pore (Figure 6e). Owing to the good water solubility of trehalose, it could rapidly dissolve and leave many pores in PLGA arrowheads after being dispersed in water. Specifically, in the appearance of PLGA arrowheads, trehalose first dissolved and was then the internalized. Based on the body fluid in the skin, a similar pore-forming process could occur after insertion of IPMNs with trehalose.

### 3.5. In Vitro Evaluation of LNG-Loaded IPMNs

**Figure 7:**
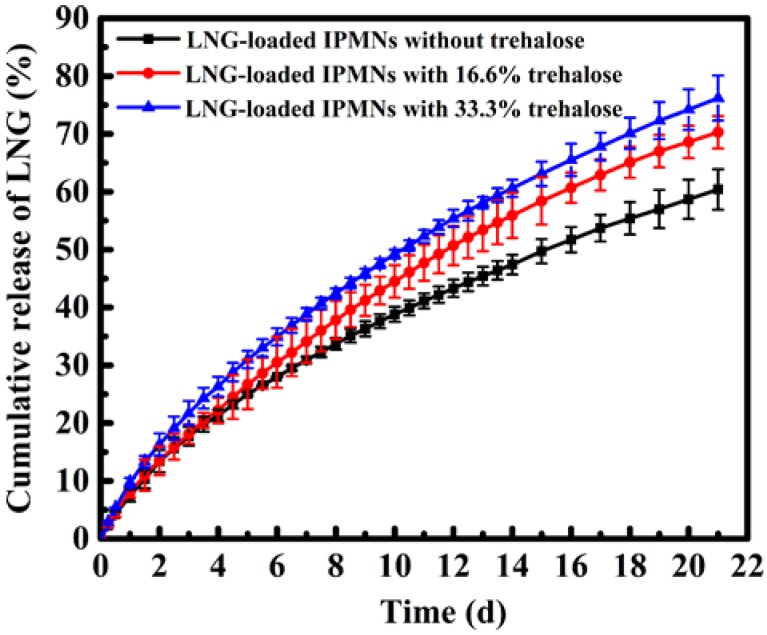
In vitro cumulative LNG release profiles from LNG-loaded IPMNs with different contents of trehalose at 37 °C (mean ± s.d., n = 3). PLGA arrowhead formulas of IPMNs all used the matrix with PLGA:LNG at a 3:2 mass ratio. LNG-loaded IPMNs without trehalose (■), with 16.6% trehalose (●) and with 33.3% trehalose (▲). The amount of LNG in one piece of IPMNs was 50 μg. Release medium was 40% polyethylene glycol 400-PBS, pH = 7.4.

The release behaviors were influenced by the polymer-to-drug ratio [14]. Figure S3 presents the in vitro LNG release curves of IPMNs with different PLGA to LNG ratios (including 7.5:1, 5:1, 3:1, and 3:2). The cumulative release of LNG increased with decreasing ratios of PLGA:LNG. When the ratio was 3:2, the cumulative release of LNG was 60.4 ± 3.5% for 21 days. Furthermore, the timescale would be more than one month if LNG was released completely. If the ratio was further decreased, IPMNs were imperfect, because the PLGA arrowheads were not fabricated successfully. To study the effects of porogen on the release of hydrophobic LNG, different contents of trehalose were added to PLGA arrowheads using a PLGA:LNG matrix at a 3:2 mass ratio. In this study, the highest trehalose content of PLGA arrowheads was controlled at 33.3%. The incomplete morphology of IPMNs was obtained (Figure S4) while the content of trehalose was further increased. The results of drug release, shown in Figure 7, indicated that the cumulative release of LNG was augmented with the contents of trehalose. For the first three days, the cumulative release of LNG from IPMNs with 0%, 16.6%, and 33.3% trehalose were 17.7 ± 1.6%, 18.1 ± 1.7% and 21.7 ± 2.1%, respectively. The burst release was caused by the dissolution of LNG crystals on the surface and near the exterior surface of PLGA arrowheads [50]. Meanwhile, the porosity of the arrowheads also aggravated this burst phenomenon [21]. Then, LNG-loaded IPMNs with 16.6% trehalose released 70.3 ± 2.8% of LNG during 21 days. In contrast, the group with 33.3% trehalose released 76.2 ± 3.9% of LNG in this same time interval. From days 4–21, the release behaviors of LNG were mainly dominated by the dissolution of drug crystals and the diffusion of LNG from the polymer matrix. The concentration gradient was the driving force of drug diffusion [1]. For the LNG-loaded IPMNs with trehalose, pores in the PLGA matrix provided more pathways, allowing the drug to pass through more easily [20]. Therefore, crystal dissolution and drug diffusion were accelerated due to the presence of pores [51]. Meanwhile, the LNG-loaded PLGA arrowheads with 33.3% trehalose significantly eroded after in vitro release for 21 days at 37 °C by contrasting with morphology of those at day 0 (Figure S5). The drug release was based on diffusion through pores, as well as PLGA erosion. Furthermore, the quicker erosion of the PLGA matrix was ascribed to the porosity, which also expedited the drug delivery [14]. The quantity and sizes of pores increased with increasing porogen content [11]. Therefore, the release rate of LNG increased, along with the increase in trehalose amount.

The in vitro transdermal release curves of LNG from IPMNs with different contents of trehalose were also studied. The results are shown in Figure S4. The cumulative transdermal amount of LNG released from IPMNs with 0%, 16.6%, and 33.3% trehalose were 46.70 ± 2.08 μg/cm^2^, 58.92 ± 2.74 μg/cm^2^, and 64.91 ± 1.25 μg/cm^2^ over 21 days, respectively. LNG is widely active used a birth control drug [30]; its minimum contraceptive dose is approximately 25 μg/day [52]. That is, 525 μg of LNG is needed for 21 days of release. For LNG-loaded IPMNs with 0%, 16.6%, and 33.3% trehalose, the sizes required for contraception for 21 days are approximately 11.24 cm^2^, 8.91 cm^2^, and 8.09 cm^2^. Compared with the size of IPMNs without trehalose, areas of IPMNs with 16.6% and 33.3% trehalose were reduced by approximately 21% and 28%, respectively. The area reduction of LNG-loaded IPMNs is more convenient for carry and use, making its use less burdensome.

### 3.6. LNG Pharmacokinetics in Rats

**Figure 8:**
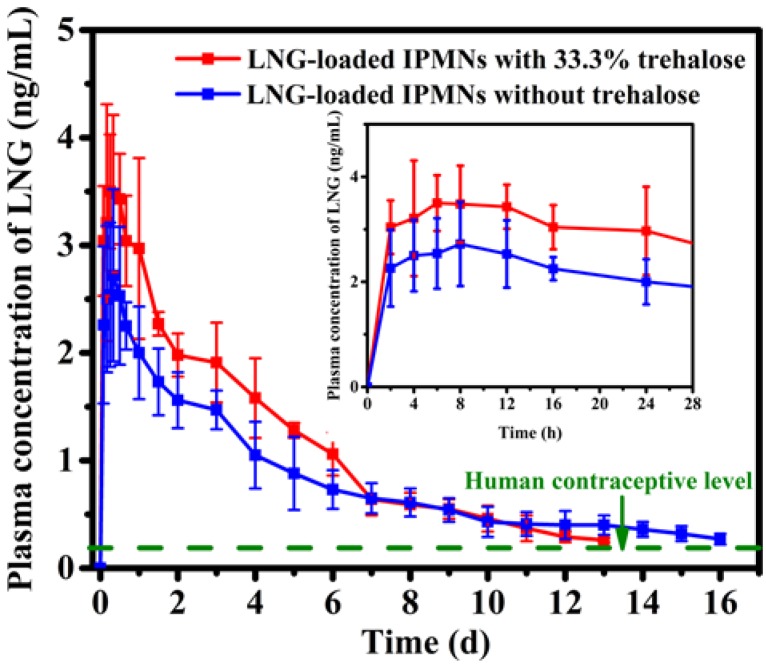
Plasma concentration of LNG versus time curves after administration of LNG-loaded IPMNs with 33.3% trehalose (■) and without trehalose (■) in vivo in rats (mean ± s.d., n = 6). The inset shows LNG concentration-time curves on the first day. The contraceptive LNG level in humans is shown by the green dashed line. The dose of LNG was 500 μg/rat.

LNG-loaded IPMNs with 33.3% trehalose and without trehalose were applied to rats to evaluate LNG pharmacokinetics. Figure 8 shows plasma concentration of LNG versus time profiles after administration. LNG plasma concentrations of both groups dropped gradually after reaching their respective peak levels. Higher C_max_ (3.5 ± 0.5 ng/mL) and shorter T_max_ (6 h) were observed in the IPMNs group with 33.3% trehalose, compared with the group without trehalose (Table 1). During the first six days, the curve of IPMNs group with 33.3% trehalose always exceeded that of the group without trehalose. The inset in Figure 8 clearly shows that the LNG plasma levels of IPMNs with 33.3% trehalose were higher than those without trehalose at each time point on the first day. After the implantation of LNG-loaded PLGA arrowheads into the skin, LNG was released from the PLGA matrix and absorbed into the bloodstream by capillaries in the dermis [53]. The porosity of PLGA arrowheads greatly accelerated the release of LNG, which led to a higher C_max_, shorter T_max_, and higher LNG plasma levels that were sustained for six days in the group of LNG-loaded IPMNs with 33.3% trehalose. After the sixth day, the plasma concentrations of both groups decreased gradually, maintaining LNG levels of between 0.25 and 0.65 ng/mL for several days. Due to the same intake dose of these two groups (500 μg/rat), the greater the LNG amount released in the early days, the less residual LNG there is in the PLGA matrix. Therefore, LNG plasma levels after treatment of LNG-loaded IPMNs with 33.3% trehalose were close to those without trehalose after the sixth day. Finally, for the group of LNG-loaded IPMNs with 33.3% trehalose, maintenance of LNG concentration beyond the contraceptive level (0.2 ng/mL [54]; the contraceptive LNG level in humans is indicated by the green dashed line in Figure 8) lasted 13 days while this level was maintained for 16 days in the LNG-loaded IPMNs without trehalose group. A shorter timescale of approximately two weeks of LNG released from LNG-loaded IPMNs with trehalose in vivo was obtained, which provided a potentially useful method for the quicker release of hydrophobic drugs from PLGA-based MNs. Additionally, semimonthly contraceptive IPMNs could add a new option for birth control.

The AUC for LNG release from IPMNs with 33.3% trehalose was obviously higher than that without trehalose (*p* < 0.05). The accelerated release kinetics of LNG could also result in a higher bioavailability of LNG for IPMNs with 33.3% trehalose than those without trehalose at the same dose. Additionally, the AUC value of subcutaneous LNG injection was 310.3 ± 58.3 ng·h/mL (Figure S5 and Table S3), which indicated that the bioavailability of LNG delivery from IPMNs with 33.3% trehalose was similar to subcutaneous LNG injection (*p* > 0.05). Importantly, the pain and fear caused by injection would not be present with IPMNs [53,55].

### 3.7. In Vivo Degradability of PLGA Arrowheads

**Figure 9:**
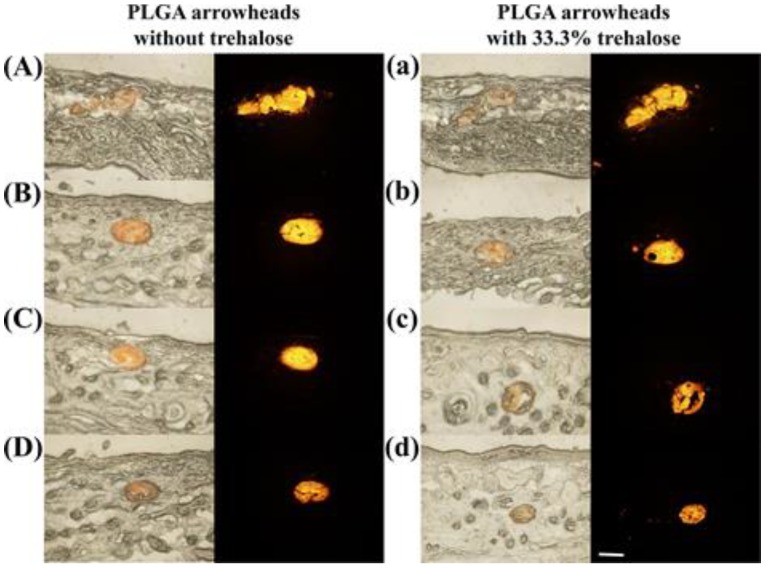
In vivo degradation of FR-loaded PLGA arrowheads without trehalose and with 33.3% trehalose. Histological sections of rat skin at day 0 (**A**,**a**), 1 week (**B**,**b**), 2 weeks (**C**,**c**), and 3 weeks (**D**,**d**) after application of FR-loaded IPMNs in vivo. For each group, the left image shows bright-field micrographs, and the right image shows fluorescence images. Scale bar, 100 μm.

To visualize the effects of trehalose on the PLGA degradation rate, FR-loaded IPMNs without trehalose and with 33.3% trehalose were inserted into rat skin in vivo. Figure 9A,a shows that FR-loaded PLGA arrowheads were left completely embedded in the skin after peeling off the IPMNs. Moreover, for these two groups, the morphologies of the PLGA arrowheads were equivalent at the beginning. Then, the PLGA arrowheads gradually eroded over time. Figure 9B–d show that the residual PLGA arrowheads with trehalose were smaller than those without trehalose at the same time points. These results indicate that the PLGA matrix with trehalose degraded quickly. At three weeks after application, the PLGA arrowheads for these two groups were not completely eroded, and remained in the skin. However, the eroded PLGA arrowheads with trehalose were visibly smaller than those without trehalose. Based on morphologies of the residual PLGA arrowheads, it could be predicted that the erosion period of PLGA arrowheads with 33.3% trehalose was approximately 4–6 weeks, and that of those without trehalose was approximately 6–8 weeks. For PLGA arrowheads with 33.3% trehalose, the porosity was caused by the dissolution of trehalose. The porous structure could result in a higher specific surface area, which expedites the degradation rate of PLGA [56]. Additionally, the PLGA degradation could contribute to drug delivery [51].

## 4. Conclusions

In this work, IPMNs were shown to be able to implant LNG-loaded PLGA arrowheads into the skin by the dissolution of a water-soluble supporting array. We studied the effect of trehalose on the release of hydrophobic LNG from PLGA arrowheads. The porous structure in PLGA arrowheads was created by the dissolution of trehalose, which was responsible for facilitating the release of LNG and PLGA erosion. Due to the porosity of PLGA arrowheads, a shorter release timescale of LNG, higher AUC, and quicker PLGA degradation were achieved through the application of LNG-loaded IPMNs with trehalose in vivo. This system is promising and will provide an effective way to modulate the time interval of hydrophobic drug release from PLGA-based MNs. However, the current study still had some limitations. For example, the in vivo release behavior of hydrophobic LNG needs to be further regulated to satisfy more patients’ needs.

## Figures and Tables

**Table 1:** Pharmacokinetic parameters of plasma LNG concentrations after administration of LNG-loaded IPMNs with 33.3% trehalose and without trehalose in vivo in rats (mean ± s.d., n = 6). Peak plasma concentration (C_max_), the time when C_max_ was reached (T_max_), and the area under the plasma concentration versus time curve from time zero to the time of the last detection (AUC_0–t_).

Group	T_max_ (h)	C_max_ (ng/mL)	AUC_0–t_ (ng·h/mL)
LNG-loaded IPMNs (with 33.3% trehalose)	6	3.5 ± 0.5	326.5 ± 49.8 (0–13 days)
LNG-loaded IPMNs (without trehalose)	8	2.7 ± 0.8	264.6 ± 39.8 (0–16 days)

## Supplementary Materials

The following are available online at https://www.mdpi.com/2073-4360/12/1/59/s1, Figure S1: (a) Stereomicroscopic image of the porcine ear skin after FR-loaded IPMNs insertion and detachment in vitro. (b) Stereomicroscopic image of FR-loaded IPMNs after removed from the porcine ear skin, Figure S2: Mechanical performance of LNG-loaded IPMNs without trehalose, with 16.6%, and with 33.3% trehalsoe. PLGA arrowhead formulas of IPMNs were all used the matrix with PLGA:LNG at a 3:2 mass ratio. The inset shows the image of porcine skin after insertion of LNG-loaded IPMNs with 33.3% trehalose, Figure S3: In vitro cumulative LNG release profiles from LNG-loaded IPMNs with different ratios of PLGA to LNG at 37 °C (mean ± s.d., n = 3). Release medium was 40% polyethylene glycol 400-PBS, pH = 7.4, Figure S4: Side view of LNG-loaded IPMNs with 38.8% trehalose. PLGA arrowhead formula of IPMNs was used the matrix with PLGA:LNG at a 3:2 mass ratio, Figure S5: Bright field micrographs of LNG-loaded IPMNs with 33.3% trehalose at day 0 (a), 21 days (b) after in vitro release at 37 °C. PLGA arrowhead formula of IPMNs was used the matrix with PLGA:LNG at a 3:2 mass ratio. Release medium was 40% polyethylene glycol 400-PBS, pH = 7.4, Figure S6: In vitro cumulative LNG transdermal amount from LNG-loaded IPMNs with different contents of trehalose at 37 °C (mean ± s.d., n = 4). PLGA arrowhead formulas of IPMNs were all used the matrix with PLGA:LNG at a 3:2 mass ratio. LNG-loaded IPMNs without trehalose (■), with 16.6% trehalose (●) and with 33.3% trehalose (▲). The amount of LNG in one piece of IPMNs was 50 μg. Release medium was 40% polyethylene glycol 400-PBS, pH = 7.4, Figure S7: Plasma concentration of LNG versus time curve after administration of subcutaneous LNG injection in vivo in rats (mean ± s.d., n = 6). The dose of LNG was 500 μg/rat, Table S1: PLGA arrowhead formulations, Table S2: The drug loading of IPMNs with different PLGA arrowhead formulations (mean ± s.d., n = 5). Note: 15% (w/w) solids composed of PLGA and LNG with different mass ratios were dispersed in NMP, Table S3: Pharmacokinetic parameters of plasma LNG concentrations after administration of subcutaneous LNG injection in vivo in rats (mean ± s.d., n = 6).
