# Supplementary material for: Exploring Trehalose on the Release of Levonorgestrel from Implantable PLGA Microneedles
Source: Polymers (Basel). 2020 Jan 1;12(1):59. doi: 10.3390/polym12010059 (PMC7023614; doi:10.3390/polym12010059)
Supplement: Supplementary file 1 [file polymers-12-00059-s001.pdf]

polymers

MDPI

Article

Exploring Trehalose on the Release of Levonorgestrel from Implantable PLGA Microneedles

Xiaoyu Zhao[1,2], Suohui Zhang[1], Guozhong Yang[1], Zequan Zhou[1,2], Yunhua Gao[1,2*]

[1] Key Laboratory of Photochemical Conversion and Optoelectronic Materials, Technical Institute of Physics and Chemistry, Chinese Academy of Sciences, Beijing, 100190, China; xyzhao@mail.ipc.ac.cn (X.Z.); suohuizhang@mail.ipc.ac.cn (S.Z.); yangguozhong@mail.ipc.ac.cn (G.Y.); zhouzequan16@mails.ucas.edu.cn (Z.Z.)

[2] University of Chinese Academy of Sciences, Beijing, 100049, China;

* Correspondence: yhgao@mail.ipc.ac.cn (Y.G.); Tel.: +86 010 8254 3581 (Y.G.)

Received: date; Accepted: date; Published: date

Supplementary Materials:

Table S1. PLGA arrowhead formulations.

Formulation	PLGA (w/w)	FR (w/w)	Trehalose (w/w based on PLGA amount)
1	14.5%	0.5%	0%
2	9.0%	0%	0%
3	9.0%	0%	33.3%
4	9.0%	0.5%	33.3%
5	9.0%	0.5%	0%

Table S2. The drug loading of IPMNs with different PLGA arrowhead formulations (mean ± s.d., n=5). Note: 15% (w/w) solids composed of PLGA and LNG with different mass ratios were dispersed in NMP.

PLGA arrowhead formulation		
PLGA:LNG (w:w)	Trehalose (w/w based on PLGA amount)	Drug loading of IPMNs (μg/piece)
7.5:1	0%	20.1 ± 0.5
5:1	0%	30.4 ± 0.7
3:1	0%	40.2 ± 0.9
3:2	0%	49.6 ± 1.5
3:2	16.6%	49.8 ± 1.2
3:2	33.3%	50.0 ± 1.4

Table S3. Pharmacokinetic parameters of plasma LNG concentrations after administration of subcutaneous LNG injection in vivo in rats (mean ± s.d., n=6).

T_{max} (h)	C_{max} (ng/mL)	$AUC_{0\text{–}t}$ (ng·h/mL)
2	36.4 ± 8.6	310.3 ± 58.3 (0–5 days)

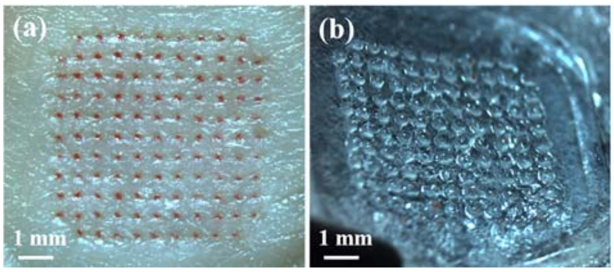

Figure S1. (**a**) Stereomicroscopic image of the porcine ear skin after FR-loaded IPMNs insertion and detachment in vitro. (**b**) Stereomicroscopic image of FR-loaded IPMNs after removed from the porcine ear skin.

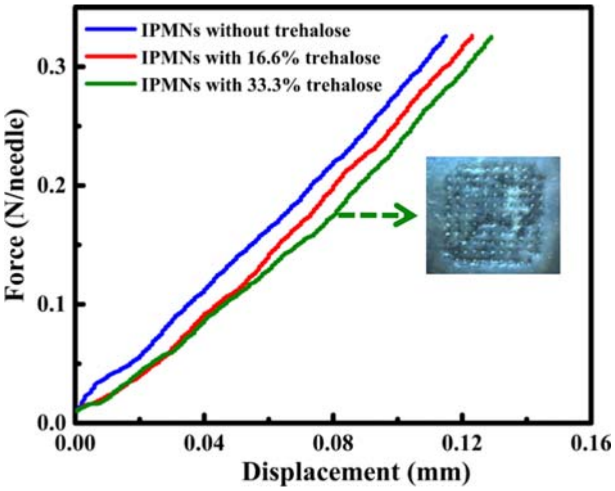

Figure S2. Mechanical performance of LNG-loaded IPMNs without trehalose, with 16.6%, and with 33.3% trehalsoe. PLGA arrowhead formulas of IPMNs were all used the matrix with PLGA:LNG at a 3:2 mass ratio. The inset shows the image of porcine ear skin after insertion of LNG-loaded IPMNs with 33.3% trehalose.

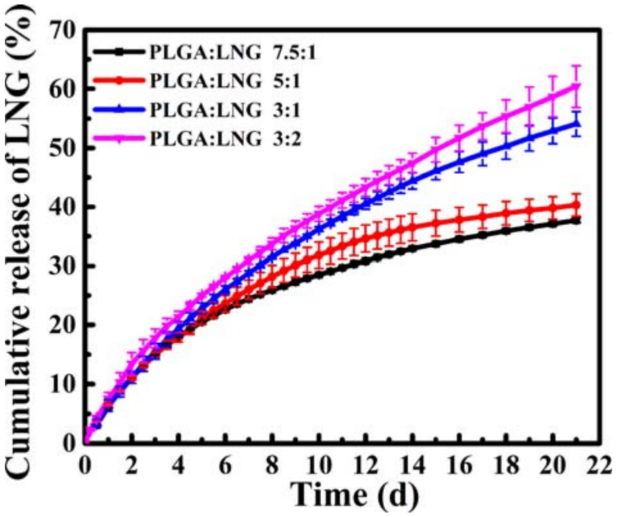

Figure S3. In vitro cumulative LNG release profiles from LNG-loaded IPMNs with different ratios of PLGA to LNG at 37°C (mean ± s.d., n=3). Release medium was 40% polyethylene glycol 400-PBS, pH = 7.4.

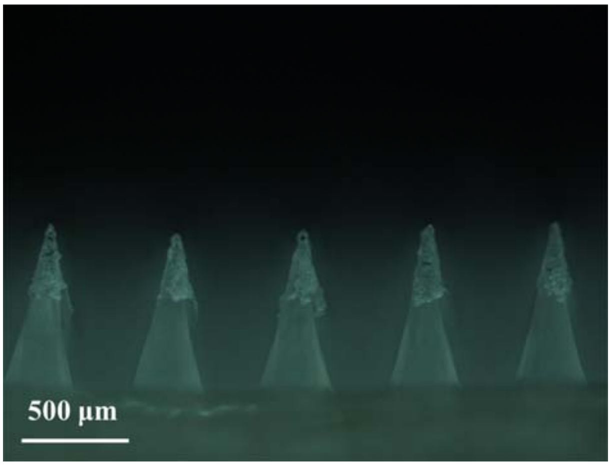

Figure S4. Side view of LNG-loaded IPMNs with 38.8% trehalose. PLGA arrowhead formula of IPMNs was used the matrix with PLGA:LNG at a 3:2 mass ratio.

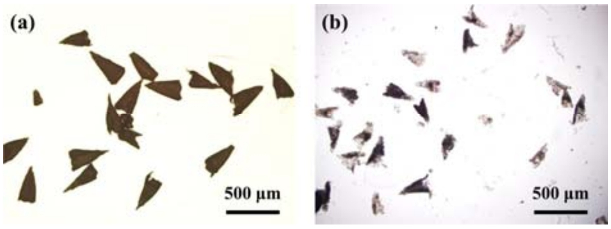

Figure S5. Bright field micrographs of LNG-loaded IPMNs with 33.3% trehalose at day 0 (**a**), 21 days (**b**) after in vitro release at 37° C. PLGA arrowhead formula of IPMNs was used the matrix with PLGA:LNG at a 3:2 mass ratio. Release medium was 40% polyethylene glycol 400-PBS, pH = 7.4.

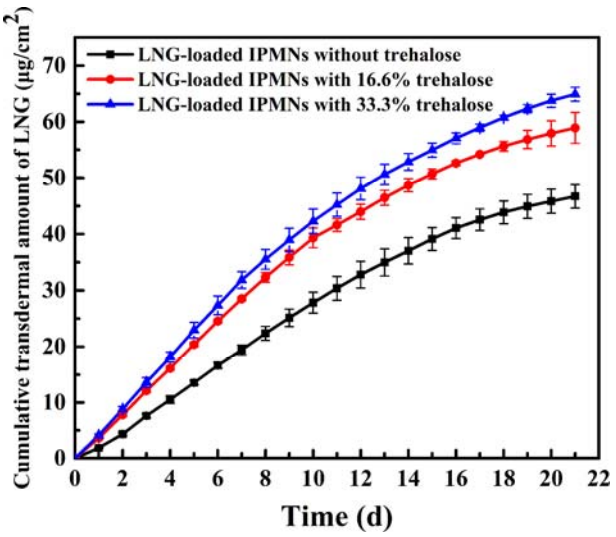

Figure S6. In vitro cumulative LNG transdermal amount from LNG-loaded IPMNs with different contents of trehalose at 37°C (mean ± s.d., n=4). PLGA arrowhead formulas of IPMNs were all used the matrix with PLGA:LNG at a 3:2 mass ratio. LNG-loaded IPMNs without trehalose (■), with 16.6% trehalose (●) and with 33.3% trehalose (▲). The amount of LNG in one piece of IPMNs was 50 μg. Release medium was 40% polyethylene glycol 400-PBS, pH = 7.4.

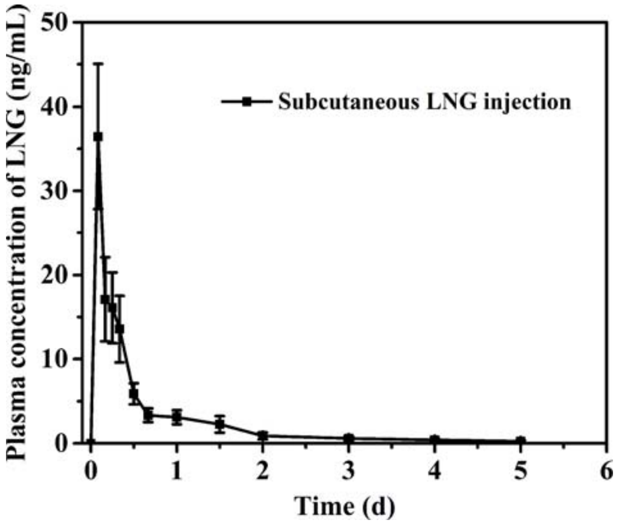

Figure S7. Plasma concentration of LNG versus time curve after administration of subcutaneous LNG injection in vivo in rats (mean ± s.d., n=6). The dose of LNG was 500 μg/rat.

© 2019 by the authors. Submitted for possible open access publication under the terms and conditions of the Creative Commons Attribution (CC BY) license (http://creativecommons.org/licenses/by/4.0/).
